# Miscellaneous

**Published:** 1891-11

**Authors:** 


### MISCELLANEOUS.

### BABIES BORN ANNUALLY.

It has been computed that between 36,000,000 and 37,000,000 babies are born into the world each year. The rate of production is, therefore, about seventy per minute, or more than one for every beat of the clock. With one one-a-minute calculation every reader is familiar, but it is not every one who stops to calculate what this means when it comes to a year's supply. It will, therefore, probably startle a good many persons to find on the authority of a well known statistician that, could the infants of a year be ranged in a line in cradles, the cradles would extend around the globe. The same writer looks at the matter in a still more picturesque light. He imagines the babies being carried past a given point in their mothers' arms, one by one, and the procession being kept up night and day until the last hour in the twelfth month had passed by. A sufficiently liberal rate is allowed, but even in going past at the rate of twenty a minute, 1,200 an hour during the entire year, the reviewer at his post would only have seen the sixth part of this infantile host. In other words, the babes that had to be carried when the tramp began would be able to walk when but a mere fraction of its comrades had reached the reviewer's post, and when the year's supply of babes was drawing to a close there would be a rear guard, not of infants, but of romping six-year-old boys and girls.

### TRANSPIRATION.

It is absolutely essential to health that the emanations from the skin pass easily through the clothing. This—which is called "transpiration"—may be interfered with by an excess of clothing, or by clothing of a very close texture. All who wear India rubber coats know how uncomfortable they cause them to feel after they have been on a short time. Ordinarily clothing will not, of course, prevent transpiration, but an excess will interfere with it; and where too much clothing is worn the same soon becomes foul, unless the outside air can freely mingle with the gases from the body and so dilute them. Some wear the thickest and heaviest undervests which they can buy, and such people are very generally the victims of frequent colds. Following the rule of light clothing they would be much safer from the dangers of exposure were they to wear two light undervests instead of one very thick and heavy.

### OUTSPOKEN.

In a recent sermon Rev. M. J. Savage, referring to "the great army of Spiritualists," said: In spite of frauds and delusions, which are only too numerous; in spite of all the "exposures" false or true; in spite of learned "explanations" of all the strange phenomena—it is still true that this army is on the increase.

Converts from science, the church, and the world are swelling their ranks. Only still more evidence of depravity, thinks the church; only another swelling toward the flood of the ever turning tide of popular superstition, thinks science. In any case, it is true the tide is rising, whatever be the cause. Scientists, philosophers, physicians, statesmen, novelists, poets, artists, jurists, people of every rank and country, are declaring their conviction that those we call the dead do live, and that they can send back proofs of both their existence and their identity.

Robert Buchanan in "Some Memories of Boyhood," says: A young Scotchman some years younger than myself, came to stay with me—Charles Gibbon, since well-known as a story writer. He was an earnest, open-hearted boy, and we lived together in great mutual happiness. We worked hard indeed (for literature is never liberally paid), and more than once sat writing, without going to bed for a fortnight at a stretch. One night he wakened up and found me crying. "What the matter?" he asked. "David Gray is dead," I answered, though I had had no word of my friend for over a week. The next post from Scotland brought me the news of David's death. "God has love, and I have faith!" were almost his last words.

A young New York girl enjoying the season in London, writes home; "Of course you have seen a good deal in the papers about Nina Kennedy, the inspirationalist, as people call her. If she keeps on as she has begun she will make all London afraid of her. She seems to know the most wonderful things about your past life and present circumstances, and all that she does is to put her fingers on your pulse and look into your eyes. I went to her the other day with Mrs.——, who was divorced you know, last year. 'You have been married?' she said to her the minute she touched her wrist. 'Yes.' 'But you are living apart from your husband?' 'Why do you think so?' 'I don't think so; I know it. Your pulse is not that of a married woman.'" We shall have no doubt before long a still further differentiation of pulses. What a field of study is opened up by the pulse of the engaged girl, and what a complicated pulse must be that of the widow about to re-marry!

### NATURAL SLEEP.

Neurosine is a never failing remedy in producing sleep without the disagreeable after effects which follow the use of opium. It is the remedy, par excellence, to relieve those addicted to the excessive use of stimulants, removing the desire for same, and toning up the nervous system. Highly recommended in cases of over exertion of mind and body.

### BE CHEERFUL.

I give you the precept for just what it is worth, as I would recommend to you to be six feet, or, at least, five feet ten, in stature. You cannot settle that matter for yourself, but you can stand up straight, and give your five feet five its full value. You can help along a little by wearing high-heeled shoes. So you can do something to encourage yourself in serenity of aspect and demeanor, keeping your infirmities and troubles in the background instead of making them the staple of your conversation. This piece of advice, if followed, may be worth from three to five years of life to you.—Dr. O. W. Holmes.

### SUBSTITUTION FRAUDS.

Public attention has recently been brought to bear on substitution by a most excellent address delivered by Mr. A. Frank Richardson, before the National Editorial Convention at St. Paul.

It is charged that all over the country there is a great system of substitution going on. There are many standard articles well known to the public through the efforts of their manufacturers who have spent fabulous sums of money in placing their merits before the masses by advertising. A large number of druggists and dealers have taken advantage of these circumstances and counterfeit the goods just closely enough to escape legal proceedings. There are several establishments which make a business of manufacturing these imitations, making them to resemble the standard article in both name and appearance of the packages. Druggists buy these, frequently having their names printed on the wrappers, and sometimes the words "our own make."

A customer going into one of these drug stores and asking for one of the well known proprietary articles he has seen so much advertised will be told by the proprietor that he has something else "just as good." The druggist then declares that it is a compound of his own and that he can recommend it, at the same time quoting a lower price than of the article asked for. The trick generally works and the purchaser goes away with probably a worthless and dangerous mixture.

### HANDLING OF CHILDREN.

It is often painful to observe how little children are handled. It is not an uncommon practice for parents and nurses to catch them suddenly by the hand or arm, and drag or hurl them over some difficult spot, such, for instance, as a mud-hole, or over a brook, if in the country; or from the steps of a tramcar to the pavement, or over some broken place in the pavement or street; or over the gutter, if in the town, without a single thought of what the consequences might be from such procedure. If parents and nurses who are guilty of such conduct will, by way of experiment, just allow themselves to be suddenly suspended by the wrist or arm, and at the same time hurled across a given space, they will have taken the first lesson in reference to the impropriety, not to say barbarous and brutal character, of such a practice.

### HINTS ON DIET.

Less meat is needed in hot weather. Fruits, vegetables, milk and eggs, all produced on the farm, all fresh each day, should form the principal part of the diet now. A good long rest at noon, and work in the cool of the day, is proper.

Sitting about in the open air evenings, beware of becoming chilled ; dangerous colds may be contracted, even in hottest weather by imprudence in this respect. Coats and shawls should always be near. Bathe for health. Cleanliness of body promotes sleep and conduces to a feeling of respectability. But for bos toy loiter about ponds and mill-dams, inhaling damp miasmatic air, may not be well.

### A FEW DON'TS FOR GIRLS.

Don't keep the fact that you are corresponding with some man a secret from your mother.

Don't let any man kiss you or put his arm about you unless you are engaged to be married to him, and even then be a little stingy with your favors.

Don't let Tom, Dick, or Harry call you by your first name, or greet you with some slang phrase.

Don't let any man believe that simply for the asking he can get "that pretty Smith girl" to go out driving with him, to accompany him to the concert, or to entertain him for an hour when he can't find any body else.

Don't write foolish letters to any body, men or women ; you never know who may see them.

Don't think that you can go untidy all day and then look very fine at night, for fine feathers do not always make fine birds.

Don't believe that you can be careless in speech or manner without its absolutely having a bad moral effect upon you.

My dear girl, it's in your own hand as to what you will be. An intelligent, charming woman or a foolish, ignorant one, and certainly if a few "don'ts" will save you from being the last, you ought not only to read and learn, but inwardly digest and practice.—*Ladies' Journal.*

### SOMETHING NEW.

The College of Physicians and Surgeons of Chicago has inaugurated a non-resident course, covering the first year of the full course. This will doubtless be largely availed of. Send for Prospectus to Dr. Bayard Holmes, Secretary, 240 Wabash Avenue.

